# Mutation-based clustering and classification analysis reveals distinctive age groups and age-related biomarkers for glioma

**DOI:** 10.1186/s12911-021-01420-1

**Published:** 2021-02-27

**Authors:** Claire Jean-Quartier, Fleur Jeanquartier, Aydin Ridvan, Matthias Kargl, Tica Mirza, Tobias Stangl, Robi Markaĉ, Mauro Jurada, Andreas Holzinger

**Affiliations:** 1 Human-Centered AI Lab (Holzinger Group), Institute for Medical Informatics, Statistics and Documentation, Medical University Graz, Auenbruggerplatz 2/V, 8036 Graz, Austria; 2 Institute of Interactive Systems and Data Science, Graz University of Technology, Graz, Austria

**Keywords:** Glioma classification, pediatric cancer, explainable artificial intelligence, XAI, Age clusters, K-Means, Random Forest, IDH1

## Abstract

**Background:**

Malignant brain tumor diseases exhibit differences within molecular features depending on the patient’s age.

**Methods:**

In this work, we use gene mutation data from public resources to explore age specifics about glioma. We use both an explainable clustering as well as classification approach to find and interpret age-based differences in brain tumor diseases. We estimate age clusters and correlate age specific biomarkers.

**Results:**

Age group classification shows known age specifics but also points out several genes which, so far, have not been associated with glioma classification.

**Conclusions:**

We highlight mutated genes to be characteristic for certain age groups and suggest novel age-based biomarkers and targets.

## Background

Incidence of cancer subtypes varies among children and adults. Malignant brain tumors are the leading cause of cancer death of younger patients, while in older cohorts it is lung and bronchus cancer [1, 2].

Gliomas are brain tumors holding grades from I to IV depending on their malignancy [3]. High Grade Gliomas (HGG) are brain tumors of grade III–IV. HGG are more likely to be found in older population, while patients suffering from the most aggressive form of gliomas, the glioblastoma multiforme (GBM), have a median age of 65 years at diagnosis [4]. Childhood gliomas more often include low-grade gliomas (LGG) [5]. Regarding the term LGG, it is recommended by WHO to distinguish between diffuse gliomas and astrocytic tumors due to the substantially biologically heterogeneous group of grade I–II gliomas [6].

There are considerable molecular differences between pediatric and adult gliomas [7]. Age-dependent heterogeneity in brain tumor subgroups such as HGG and LGG differences have been described [8]. So far, there are several studies on molecular features [9–11] within pediatric or elderly patients, however, a classification involving age specifics has not been included in established schemes.

Therapy-relevant glioma classification depends on the knowledge of underlying molecular variations [12, 13]. The conventional classification was updated in 2016 and is based on gene variations. These include, primarily, codeletion of chromosomal arms 1p and 19q, and the genetic status of isocitrate dehydrogenase 1 (IDH1) [13]. Further mutations are described for Alpha thalassemia/mental retardation syndrome X-linked chromatin remodeler (ATRX) [14], tumor protein P53 (TP53) [15], telomerase reverse transcriptase (TERT) [16], H3 histone family member 3A (H3F3A) and histone cluster 1 H3 family member B or C (HIST1H3B/C) [17], B-Raf proto-oncogene, serine/threonine kinase (BRAF) [18] and KIAA1549-BRAF fusion [19], deletions of cyclin dependent kinase inhibitor 2A or 2B (CDKN2A/B) [20], fusions of RELA proto-oncogene, NF-KB subunit (RELA) [21], catenin-beta 1 (CTNNB1) referred to the group of wingless-type MMTV integration site family (WNT) [22], or PTCH and SUFU within sonic hedgehog signaling molecule (SHH)-activated subgroup [13, 23].

Over time, brain tumor classification systems have been and are, still, evolving [24]. Molecular signatures in adult gliomas have been explored and show certain subtypes in dependence on age [25, 26]. By using graph analysis on existing data we highlighted disturbed signaling components in brain cancer subtypes of gliomas [27]. Information exists on prominent mutations within gliomas that suggests different biomarkers for specific age groups [28, 29]. Further, alterations have been shown to be prevalent for specific age groups by the comparison of older and young adults [30].

Some tumors primarily occur in children, such as diffuse midline gliomas with their molecular feature of mutated H3F3A or HIST1H3B/C [31]. Pilocytic astrocytomas are common for pediatric but not elderly patients and frequently exhibit BRAF mutations and fusion transcripts [32]. Pediatric HGG frequently include PDGFR-$\alpha$ amplification different to the adult equivalent [33]. And gliomas from younger children rarely exhibit IDH mutations [34].

In spite of medical advances in cancer diagnosis and treatment, for instance, GBM treatment remains to be mostly the same (old) approach across all ages, surgery followed by radiotherapy and only occasionally more targeted chemotherapy [35]. Still, a well-tolerated therapy by adults may not be likewise applicable for a pediatric patient due to the ongoing brain development.

The older population can also be subdivided into an adult group and patients with a more advanced age. Thereby, elderly show different clinical pictures, such as larger tumor mass and distinct prognostic biomarkers [36]. The elderly population commonly refers to patients older than 65 or 70 years of age, while the term “elderly” is defined as a specific age threshold. This threshold, however, varies with geographical, social, and cultural factors [37].

Overall, novel biomarkers of brain tumors will be used for more detailed diagnostics, prognosis, therapy response control as well as targets for anti-cancer therapy towards personalized medicine [38]. So far, various targets within the signaling cascades of growth factor receptors, cell cycle, angiogenesis, antitumor immune responses and epigenetic modulators have been investigated for therapy [39]. In general, cancer signaling in glioma is predominated by angiogenesis-related pathways involving MAPK, VEGF and EFGR [40]. There are several therapeutics targeting for instance VEGF, EGFR, PDGFRa, PTEN, MDM2 [38]. Still, the heterogeneous intra-tumor microenvironment demands for new strategies. Furthermore, meaningful biological subgroups are necessary to guide the design of future clinical trials [41].

We use an explainable artificial intelligence (XAI) method, i.e. SHAP, on clinical and gene mutation data to classify and explain age-related subgroups within various gliomas.

**Fig. 1:**
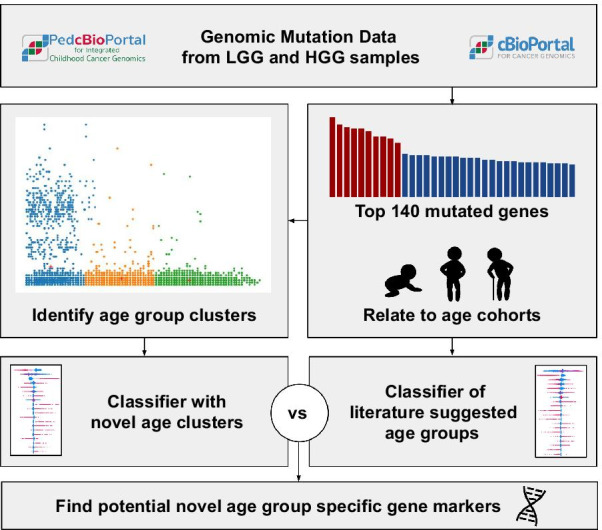
Graphical abstract

## Methods

### Data and preprocessing

The graphical abstract is shown in Fig. 1. We use data from glioma samples, including both LGG and HGG, out of 18 different projects from pedcbioportal [42, 43] via https://tinyurl.com/y5d8gubl and of 5 more projects from cbioportal via https://tinyurl.com/y2s2ogez. Both web-portals offer clinical data such as age as well as mutation details. Clinical data can be obtained through the “download” option in both web-portals. The column “mutated genes” within the overview of the web user interface (UI) can be further used to download mutation details. To overcome the query limit of max 167 different gene IDs, we further sorted the exported clinical data file by mutation count and selected only those genes that have $\ge \,2\%$ mutations. Thereby, we limited the query to the 140 top mutated genes. This list contains genes with highest mutation frequency, preceded by TP53, followed by TERT, then IDH1, etc. The 140 genes are provided as list of gene symbols as additional file 1 and on https://github.com/radiance/glioma-mutations-xai/. Filtered mutation data can be downloaded by querying the selected 140 gene names within the 18 projects from pedcbioportal as well as the 5 selected studies from cbioportal, each specified as link above.

Queried genes in pedcbioportal’s projects’ data are altered in 4210 (77%) of queried patients and 4614 (77%) of queried samples. Queried genes in cbioportal’s provided projects’ data are altered in 3032 (96%) of queried patients and 3165 (96%) of queried samples.

We extracted those columns that are relevant for clustering and classification including sample id, age and mutation count. We removed duplicated rows (such as from pbta_all and plgg_cbttc that contain parts of the same samples). We further processed the different studies’ columns by merging similar columns. Labels for clinical metadata concerning age can vary from capitalized “Age” (phgg_jones_meta_2017) and uppercase “AGE” (pbta_all, pbta_pnoc, phgg_cbttc), or “Diagnosis Age” (lgg_tcga, lgg_ucsf, gbm_tcga). We thereby excluded samples with empty or incomplete information on age as well as recurrence samples or duplicates.

We further processed mutation data. The value “na” stands for 0 mutations. Any other string represents a mutated gene. If there is a blank between characters, there are multiple mutations listed within the field. “Mutation strings” per gene can be found in the mutations.txt file. Mutation details on amino acid-changes within the specified genes are included.

Most studies provide age data as integer values. Therefore, a few samples with floating point numbers were rounded to be comparable to other integer values. We removed samples without suitable age information. Merged, filtered and reduced data covers only 2894 sample lines of 14 different projects with an age range from 0 to 90, a mutation count from 0 to 14063 and the several mutation types according to the 140 selected mutated genes. Due to this filtering process of incomplete data, 87 genes remain of the previously 140 selected genes.

**Fig. 2:**
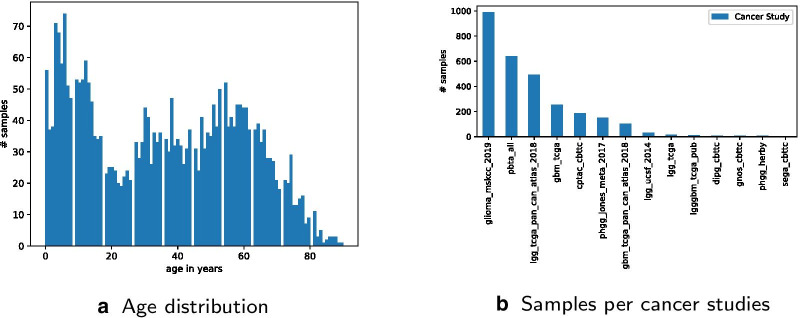
Glioma sample data distribution

**Fig. 3:**
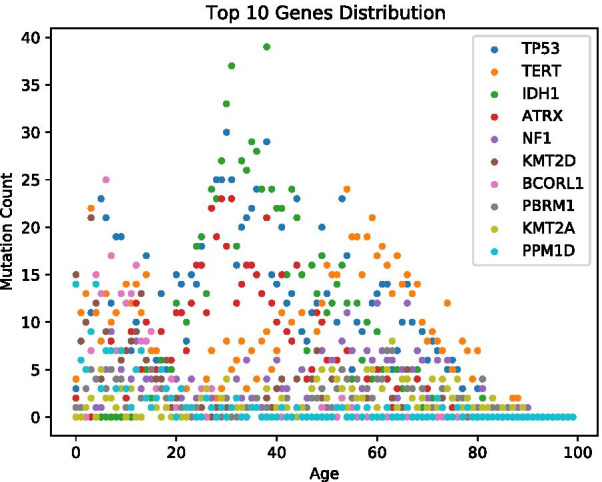
Top 10 mutated genes distribution

Age distribution is visualized in Figs. 2a and 3. Sample count per study distribution is visualized in Fig. 2b. Processed data is available as additional file 2 and on https://github.com/radiance/glioma-mutations-xai/.

### Workflow

Both clustering and classification were used to explore age-related differences in glioma diseases. We took an XAI approach to compare conventional age groups and to explore possible new age groups. Within the first classification approach the following age groups were assumed:

- Age group 0: age below 19
- Age group 1: age 19 to 70
- Age group 2: age greater than 70

We compared classifier performance by using means and standard deviation from stratified k-fold cross-validation. We selected a Random Forest approach, the best algorithm according to results from the classifier comparison, shown in Table 1. We selected the top 20 mutated genes as features. To better explain classifier results we applied SHAP (SHapley Additive exPlanations) [44] to summarize the effects of all the selected features. In parallel, we started a separate clustering approach to explore possible novel age groups. We applied a K-Means algorithm. We further used XAI principles and visually analyzed each step. Final clustering results are visualized in Fig. 5.

**Table 1:** Model comparison for classifying traditional and updated age groups (bold: best results)

	Ages 0–18, 19–70, 70+		Ages 0–22, 23–48, 48+		Ages 0–9, 10–26, 27–50, 50+	
	Mean	SD	Mean	SD	Mean	SD
**Random Forest**	**0.792226**	0.018431	**0.709313**	0.035924	**0.580960**	0.034479
Linear Discriminant	0.733046	0.028101	0.671703	0.031654	0.545130	0.031640
K Neighbors	0.738271	0.027061	0.603038	0.037627	0.473873	0.020474
Decision Tree	0.758096	0.012999	0.683384	0.031697	0.552452	0.033747
Gaussian Naive Bayes	0.225052	0.018959	0.500274	0.052508	0.358057	0.059829
C-Support Vector	0.718389	0.015940	0.561992	0.038804	0.445800	0.027187
Logistic Regression	0.750756	0.019220	0.665224	0.035580	0.549440	0.029133

**Fig. 4:**
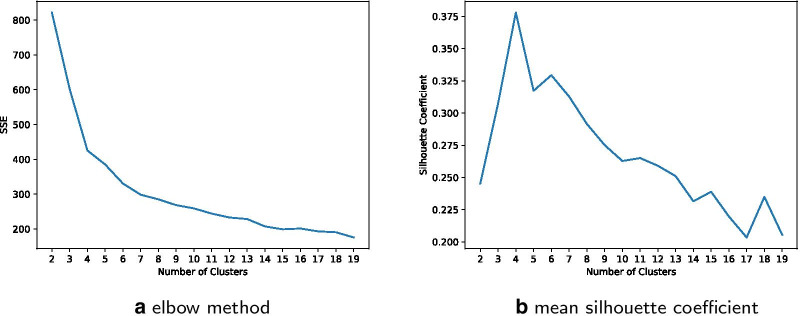
Performance functions for different number of clusters

**Fig. 5:**
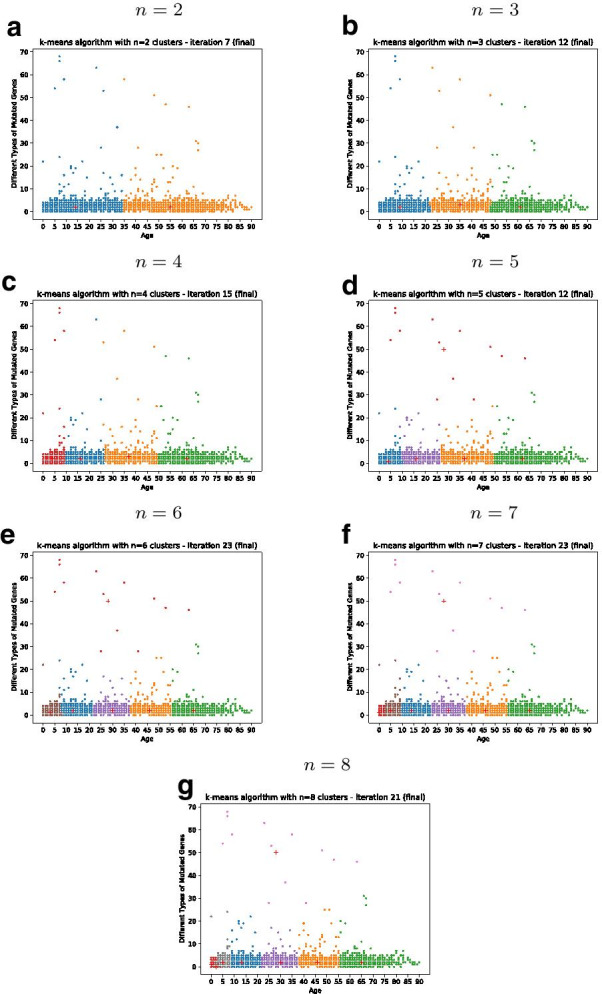
Results from gene-based clustering of age groups with different number of clusters *n*

We applied the clustering for the number of clusters to $n={2,3,4,5,6,7,8}$, as suggested by the elbow method and the silhouette coefficient, shown in Fig. 4.

Based on the visual clustering results, shown in Fig. 5, we repeated the first classification approach adapted to the three age groups as well as the four groups accordingly.

### Implementation

We implemented both a clustering as well as a classification algorithm in Python. Source code for both clustering as well as classification is available on https://github.com/radiance/glioma-mutations-xai.

#### Clustering

Clustering is based on a K-Means algorithm using Scikit-learn [45]. We further used the python libraries Pandas [46], Numpy, Matplotlib and Seaborn for data processing and visualizing results.

#### Classification

We used Scikit-learn [45] for implementing the classifiers. We used Pandas [46] for data structuring and manipulation. SHAP (SHapley Additive exPlanations) [44] was used for explainable artificial intelligence (XAI) results. We processed data and visualized the results using SHAP, Pandas and Matplotlib library.

For comparison, we repeated the classification using suggested age groups from clustering.

## Results

### Glioma sample disposition across age and clustering of age groups

Age groups are defined by using gene mutation data collected from various glioma projects. Distribution metrics of downloaded samples are shown in Figs. 2a, b and 3. Raw data included an overall of 9264 data rows, with 5961 from the studies selected via pedcbioportal and 3303 via cbioportal. After first filtering and merging data, the comma-separated values (csv) file included only 5478 data rows. The other 3786 were removed due to incompleteness and/or inappropriateness of available metadata. The column mutation count is available for only 5628 out of 6396 (768 samples not available and/or 0). This means that the 140 queried genes are altered in only 77% of selected data. The overall mutation count is not available for all samples and non-uniformly distributed over age. On the one hand, this can be explained by the fact that web portals limit query size, at least via the web user interface we used. On the other hand, pedcbioportal offered more resources on children than adult patients. Therefore, we added additional adult samples from cbioportal in order to have a more balanced age distribution. Still, we find a higher number of different mutated genes in younger patients. Finally, we excluded samples with empty or incomplete information on age and/or mutation count as well as recurrence samples and duplicates. After data cleansing, 2894 samples are left for clustering and classification experiments.

The number of clusters *n* of the K-Means algorithm can be adjusted. By computing both the sum of the squared error (SSE) as well as the silhouette coefficient, shown in Fig. 4, cluster numbers of $n\le 8$ are suggested. Different clustering results for $n={2,3,4,5,6,7,8}$ are shown in Fig. 5. The K-Means clustering for $n=3$ clusters reveals three distinctive age groups after multiple iterations:

- Class 1: age below 23
- Class 2: age 23 to 48
- Class 3: age greater than 48

The K-Means clustering for $n=4$ clusters reveals the four distinctive age groups:

- Class 1: age below 10
- Class 2: age 10 to 26
- Class 3: age 27 to 50
- Class 4: age greater than 50

Figure 5 shows a cluster number $n>4$ to show higher dissimilarity within at least one cluster (the red group in $n={5,6,7,8}$). In case of $n={5,6,7,8}$ there is at least one cluster distributed over a wide range of age.

Figure 14 and Table 2 illustrate top mutated genes of age groups from conventional and updated classes. The chart illustrates several genes associated with age. For instance, H3F3A, AHNAK2, SOX1, SUSD2 and KMT2C are most frequently mutated in young age classes. PIK3CA and TERT are upon top mutated genes within adult samples and RYR2 mutations are more frequent within older adults.

**Table 2:** Age class-specific top mutated genes: top 20 mutated genes within traditional or updated age groups from clustering within selected glioma projects

0–9	0–18	0–22	9–26	18–70	23–48	26–50	48+	70+
TTN	TTN	TTN	TP53	TP53	IDH1	IDH1	TP53	TP53
AHNAK2	TP53	TP53	ATRX	IDH1	TP53	TP53	PTEN	PTEN
TP53	AHNAK2	AHNAK2	IDH1	ATRX	ATRX	ATRX	TTN	TTN
AHNAK	H3F3A	AHNAK	TTN	PTEN	CIC	CIC	EGFR	TERT
H3F3A	AHNAK	H3F3A	AHNAK2	TTN	TTN	TTN	IDH1	EGFR
MUC17	FLG2	ATRX	H3F3A	TERT	TERT	TERT	NF1	NF1
MUC16	MUC17	FLG2	FLG2	EGFR	NOTCH1	PTEN	MUC16	PIK3R1
FLG2	MUC16	MUC17	NF1	CIC	PTEN	PIK3CA	PIK3CA	PIK3CA
PHLPP1	PHLPP1	MUC16	ERBB2	NF1	PIK3CA	NOTCH1	PIK3R1	MUC16
OBSCN	RAMP2	OBSCN	BRAF	PIK3CA	NF1	NF1	FLG	RB1
KMT2D	ATRX	NF1	RAMP2	MUC16	FUBP1	FUBP1	TERT	MUC17
SUSD2	KMT2D	RAMP2	MUC16	NOTCH1	EGFR	EGFR	CIC	PCLO
SOX1	OBSCN	KMT2D	AHNAK	PIK3R1	KMT2D	KMT2D	RYR2	RYR2
NF1	NF1	PHLPP1	MUC17	FUBP1	MUC16	MUC16	ATRX	IDH1
ATRX	BRAF	BRAF	CIC	KMT2D	SMARCA4	SMARCA4	RB1	FLG
KMT2C	SUSD2	IDH1	KMT2D	FLG	PIK3R1	PIK3R1	PCLO	USH2A
RAMP2	SOX1	KMT2C	TEX13D	RB1	ARID1A	ARID1A	SPTA1	CIC
SVIL	KMT2C	SUSD2	KMT2C	RYR2	RB1	RB1	LRP2	PDGFRA
MKI67	TEX13D	SOX1	CTNNB1	LRP1	BCOR	RYR2	MUC17	ATRX
ISM2	SVIL	TEX13D	PIK3CA	SMARCA4	NOTCH2	ATM	PKHD1	HMCN1

### Classification of age-related mutation data among gliomas

Selected age groups are compared and classified by their gene mutation signatures. At least three age groups can be distinguished from incidence reports and further studies [47, 48]. Therefore, the first classification approach is based on conventional age groups from 0–18, 19–70 and 70+.

The comparison of classifier performances suggests a Random Forest algorithm, as shown in Table 1, resulting in the best mean and standard deviation (SD). The first classification approach with 0–18, 19–70, 70+ has an accuracy of 78.41% and shows important features for age classes.

Adapting the classifier regarding the younger group to 0–22, 23–70, 70+ the accuracy drops minimally to 78.07%. Adapting the classifier regarding the older group to 0–18, 19–48, 48+ the accuracy drops to 73.58%. The adapted classifier with both groups adapted to 0–22, 23–48, 48+, as suggested by clustering results, has again a minimal lower accuracy of 72.54%. Adapting the classifier to the four suggested classes 0–9, 10–26, 27–50, 50+ lowers the accuracy further to 59.24%. An increased range of the adult age group, such as for 0–9, 10–18, 19–70, 70+, increases the accuracy to 69.08%. The use of two clusters ranging from 0–34 and 34+ leads to a classifier accuracy of 75.82%.

**Fig. 6:**
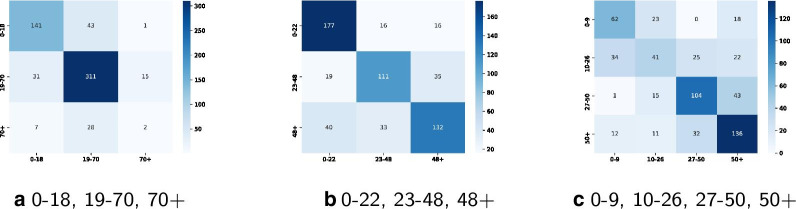
Comparison of classifier confusion matrices, showing classifier performance (darker means better prediction)

Figure 6 shows that the updated 0–22, 23–48, 48+ classifier results in a lower number of correct predictions than the first classifier with 0–18, 19–70, 70+ (420 versus 454 from overall 579). The classifier in case of four age groups shows 343 correct predictions out of 579. By comparing Tables 1 and 3 it is also shown that computed clusters are not improving the classifier’s overall performance but have impact on age group specifics. Table 3 shows precision and recall scores for the classifier versions 0–18, 19–70, 70+ and 0–22, 23–48, 48+ and 0–9, 10–26, 27–50, 50+. Comparing the youngest age group, both 3 age groups classifiers show similar results, while the 0–18, 19–70, 70+ classifier suits the middle group better, and the updated version with 0–22, 23–48, 48+ classifier performs well for the older age group. Predicting age group 50+ works best with the 0–9, 10–26, 27–50, 50+ classifier of four age groups.

**Table 3:** Performance report for classifier with traditional and updated age groups (bold: highest performing age group compared to other age groups)

Age classes	id	Precision	Recall	f1 score
**0–18**	1	**0.79**	**0.76**	**0.77**
**19–70**	2	**0.81**	**0.87**	**0.84**
70+	3	0.11	0.05	0.07
**0–22**	1	**0.75**	**0.85**	**0.80**
23–48	2	0.69	0.67	0.68
**48+**	3	**0.72**	**0.64**	**0.68**
0–9	1	0.57	0.60	0.58
10–26	2	0.46	0.34	0.39
26–50	3	0.65	0.64	0.64
**50+**	4	**0.62**	**0.71**	**0.66**

**Fig. 7:**
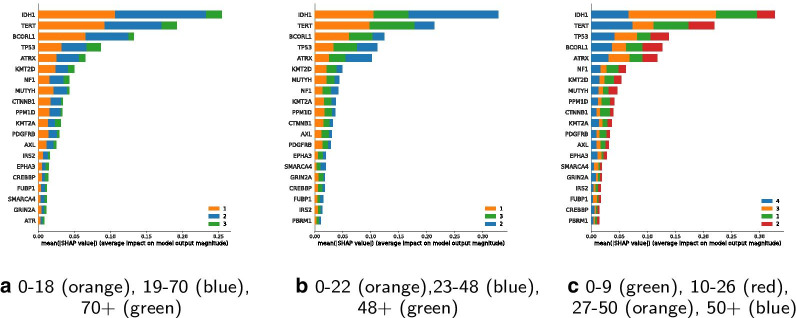
Comparison of classifier features (classes sorted by performance)

Feature importance of the classifiers 0–18, 19–70, 70+ and 0–22, 23–48, 48+ are shown in Fig. 7.

IDH1 and TP53 stay most important for classification among all classification schemes. There is a shift in importance of some other features and their association with individual age groups is changed. TERT, for instance, is highly important for the middle age group from traditional classes, its importance is shifted to the older adults from the updated classes. MUTYH has a smaller importance on the updated middle age class. So far, MUTYH mutations are infrequent and have been shown in pediatric patients to increase risk of malignant brain tumors [49].

The SHAP summary plot for the four classes 0–9, 10–26, 27–50, 50+ shows feature importance for the classification of the four suggested age groups from the clustering results. It is indicated, that IDH1 is most important for the age group of 27 to 50. TERT is most important for age group 50+.

**Fig. 8:**
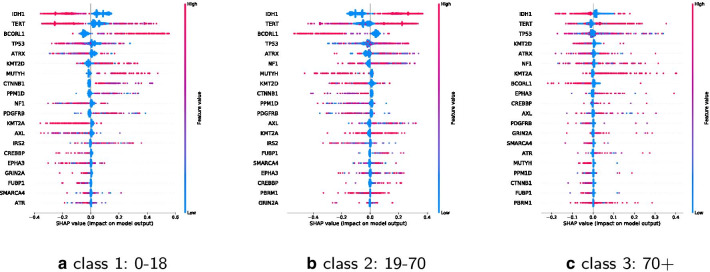
Impact of the top 20 features for each of the classes 0–18, 19–70, 70+

**Fig. 9:**
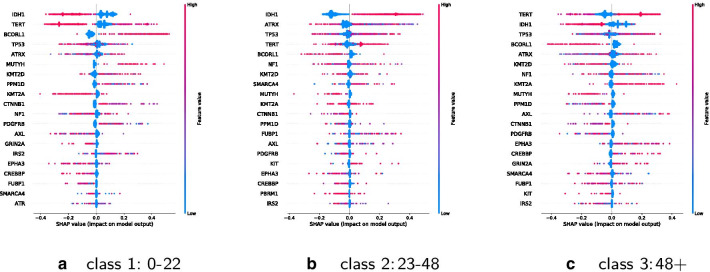
Impact of the top 20 features for each of the classes 0–22, 23–48, 48+

Figures 8 and 9 show SHAP values for the top 20 features for each class separately. A positive SHAP value increases the prediction, a negative value decreases the prediction. Features are ranked in descending order. X-axes positions refer to low up to high impact on prediction. Dots are stacked on the y-axes and refer to the concentration or respective amount of observations for a shap value. The color shows whether a shap variable is high (in red) or low (in blue) for an observation.

IDH1 has a negative impact on class 0–18 and 0–22, a positive one on class 19–70 and on 23–48, and a negative on 48+ and 70+. BCORL1 has a positive impact on classes 0–18 and 0–22 and a negative on the classes 18–70, 23–48, 48+ and 70+. KMT2D has a positive impact on young and a negative one on older age classes, whereas a high value of KMT2A has a negative impact on young and a positive on older age classes. Many other features are ambiguous.

### Comparison of HGG and LGG classifications

We further filtered data on LGG and HGG, respectively. Only a small subset of the data can be used for this comparison of subtypes. This is due to the fact that most studies contain general glioma data. Only a few studies explicitly contain either LGG-specific samples (lgg_tcga, lgg_tcga_pan_can_atlas, lgg_ucsf_2014, plgg_cbttc) or HGG-specific samples (gbm_tcga, gbm_tcga_pan_can_atlas, phgg_cbttc, phgg_herby, phgg_jones_meta_2017).

**Fig. 10:**
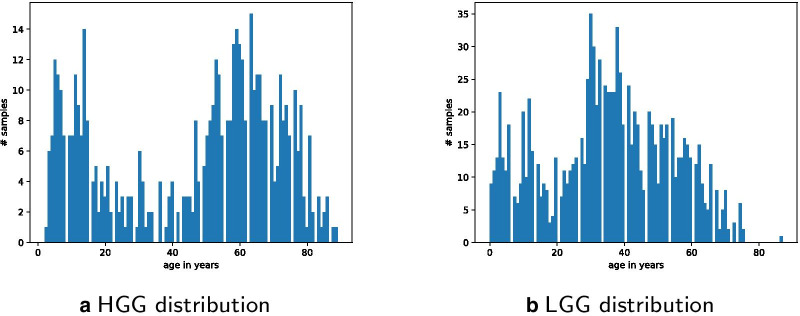
LGG and HGG specific sample data distribution per age

LGG specific data rows are 1047, HGG are 511. Figure 10 shows the distribution of data on LGG and HGG filtered samples. The prediction of the HGG classifier for the three age classes 0–18, 19–70 and 70+ has an accuracy of 67.96%, and the accuracy for LGG is 93.33%.

**Fig. 11:**
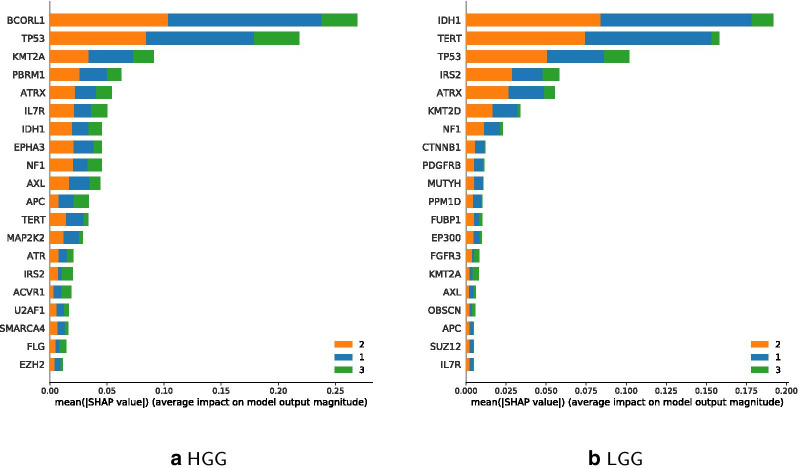
Comparison of Classifier feature importance for age classes 0–18 (blue), 19–70 (orange), 70+ (green); classes sorted by performance

**Fig. 12:**
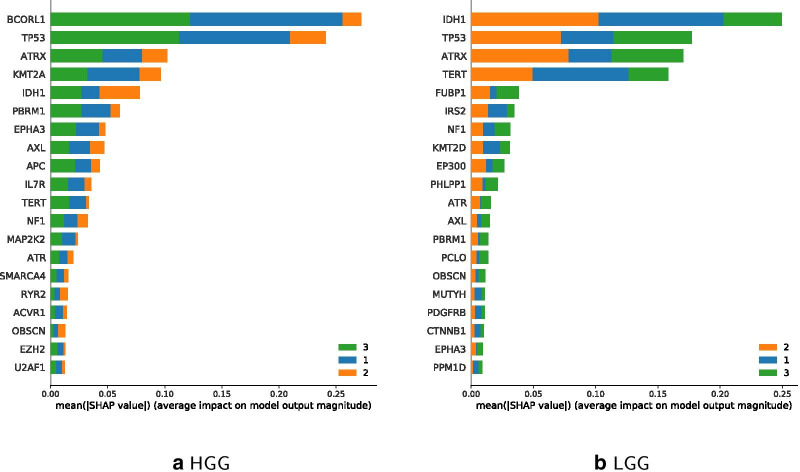
Comparison of updated classifier features for age classes 0–22 (blue), 23–48 (orange), 48+ (green), classes sorted by performance

The prediction accuracy of the updated HGG Classifier is 77.67% and for LGG 73.33%. So, the updated version performs better for HGG, while the more traditional age classes perform better with LGG-filtered data. Figure 11 shows IDH1 and TERT to be most relevant in LGG for the younger and middle age class, while BCORL1 being more dominant in HGG. Feature importance of updated classes to classify LGG and HGG are shown in Fig. 12, which highlights IDH1 as important feature for classifying the HGG younger and middle age class, and BCORL1 is more dominant for classifying the LGG younger and middle age class.

**Fig. 13:**
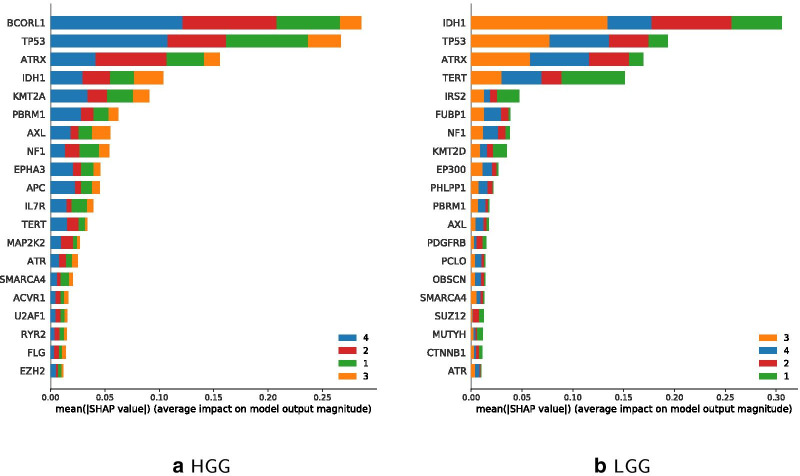
Comparison of updated classifier features for age classes 0–9 (green), 10–26 (red), 27–50 (orange), 50+ (blue); classes sorted by performance

**Fig. 14:**
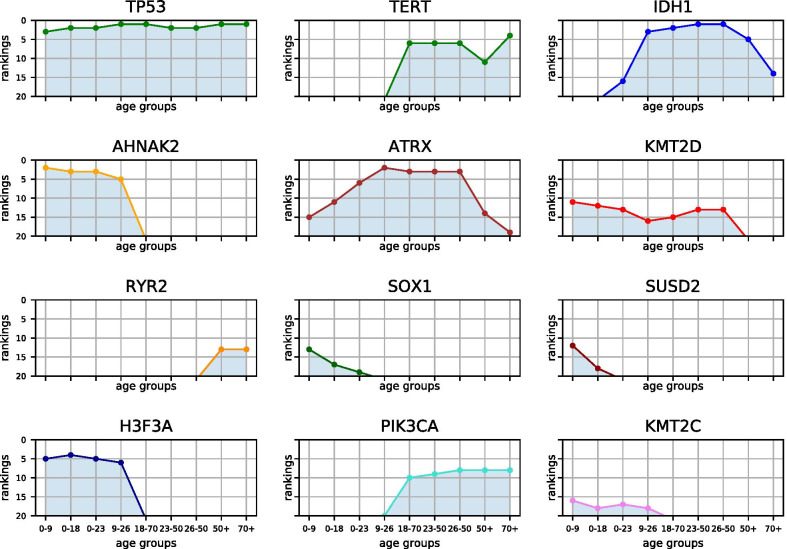
Distribution of top mutated genes within various age groups: examples from top 20 mutated genes among selected age groups of children, young adults and elderly patients suffering from glioma

Figure 13 shows estimates for classifier feature importance of classifying the 4 suggested age classes 0–9, 10–26, 27–50 on each HGG and LGG filtered data. When using $n=4$ different instead of the updated $n=3$ classes, IDH1 remains a dominant feature for age class 27–50 regarding LGG. TERT is the most important feature for classifying the youngest age group 0–9 regarding LGG. Regarding HGG, BCORL1 becomes more important for age class 50+. Comparing Feature importance for the four age classes in Figs. 12 as well as 13 shows that ATRX is less important for the youngest age group 0–9.

Figures 11, 12 and 13 further illustrate comparable importance of ATRX for all classifiers. ATRX functions as tumor suppressor and is involved in p53 signaling [50]. It has been negatively associated with TERT mutations [51]. TERT is among top 4 mutated genes in LGG and less important in HGG classes. IDH1 is the most important gene mutation succeeding TERT within LGG classification using the traditional age classes, and remains important in updated classifiers. In case of HGGs, IDH1 holds only 7th place, and in updated age classes 5th and 4th. BCORL1 is involved in tumor progression and respective mutations occur in HGGs and LGGs [52]. Still, BCORL1 is relevant for classification of HGG only. KMT2 proteins occur both in HGG and LGG under top 10 features in all classifiers. Thereby, KMT2A appears in top 5 most important features in HGG, whereas KMT2D under top 8 in LGG.

## Discussion

The main idea is to use classification as well as clustering to explore age-related differences in glioma diseases in order to find possible novel age group-specific biomarkers. We already highlighted top ten mutated genes within pediatric glioma samples from data amongst several pediatric resources, namely BRAF, TP53, KIAA1549, H3F3A, ATRX, IDH1, CDR2, PIK3CA, NF1, C17ORF47, in this order regarding mutation frequency [28]. The summary of all selected projects from pedcbioportal and cbioportal indicate age-specific mutation frequency for several genes.

Prognostic and therapeutic biomarkers for brain cancers differ between patients depending on their age. Genetic alterations in brain tumor samples show distinct gene mutation signatures related to age groups and may support the identification of novel biomarkers.

Glioma classification could be updated according to age-groups in relevance to diagnostics, therapy possibilities and clinical decision-making. We present unusual age groups for glioma classification based on gene mutation signatures. Therefrom several genes emerge as characteristics for specific age classes. The view on top ranked mutated genes in distinct age groups highlights differences in regard to diagnostics. Some genes relevant to one group could be irrelevant to another group but a previous unimportant gene could emerge as a major biomarker.

Representative age clusters disclose gene mutations as age-specific biomarkers. The clustering algorithm depicted several distinct groups but also some adjacent and marginally overlapping clusters. In case of three age clusters there is a young group up to 22 years and the middle group up to 48 years. Some sample points within the region of cluster borders may be falsely allocated. This problem would be of less importance if age was calculated in days or months instead of years.

Regarding classification performance there are several optimization possibilities. First, the higher the sample number within an age class, the higher the classifier’s accuracy. Including a higher number of data samples will improve the accuracy. Regarding data quality, even a great portal as pedcbioportal depends on data providers to allocate comparable study data. Therefore, improving the quality of clinical data, such as consistently providing more details on age at diagnosis, would further improve accuracy and specificity. Moreover, identifying cancer subtype-specific top mutated genes and using these instead of the 140 selected gene symbols, may also improve the classifier’s performance.

By comparing classifier performance, as can be seen in Fig. 6, the updated classifier for the middle group is worse rated, compared to the younger and the older age groups, which perform better. The quality of performance is demonstrated by the count in the diagonal from top left to bottom right. A higher count refers to better performance.

It can be observed, that in the first classifier version with age classes 0–18, 19–70, 70+, the middle group performs best. Nonetheless, if the goal is to detect members of one specific class rather than having a minimal better overall accuracy, one may have a closer look at the feature importance comparison, as shown in Fig. 7.

Some features are more important for specific classes. Exemplary, IDH1 is less important for the oldest age group. This can be explained insofar as IDH1 is known to be most common in LGG [53] and a substantial number of samples are from patients with LGG. By comparing both plots of classifier feature importance, shown in Fig. 7, one may observe certain changes in feature rankings.

Class-specific top mutated genes point out several genes in correlation to age. In case of young age groups, there are some genes implicated to other cancers whose role in glioma has to be elucidated, yet, like AHNAK2 and SUSD2 [54, 55]. SOX1 has been implicated with glioma, while SOX2 has been depicted as unfavorable prognostic marker [56, 57]. Older age groups include well-known biomarkers such as TERT, PTEN and NF1 which are not within the most frequent mutated genes within younger patients [58, 59].

The comparison of high and low grade gliomas further depicts several gene mutations distinct to glioma grades. Top 20 mutated gene lists from classification experiments on HGG or LGG data include either KMT2A in HGG, or KMT2D in LGG in all classification modes. Additionally, KMT2A was negatively implicated in young age classes and positively implicated in older age groups. KMT2D was inversely associated. Such observations can help elucidate the role of KMT2 proteins in tumor progression and as driver or passenger mutations in future aspect of clinical implication for the Lysine Methyltransferase 2 family [60]. Within the top 20 list of HGG age group classification several genes are highlighted that have not been associated with glioma classification, yet. Depending on their shap value they could become important for a defined age-group. Future studies will elucidate a possible clinically relevant role.

Possible misconceptions when quantifying feature relevance using Shapley values were described by [61]. Therefore, future work should further test SHAP explanations with different stakeholders [62, 63].

Amongst the top 20 mutated genes of pediatric-only patient data from pedcbioportal, there are e.g. LRP1 and HSPG2 that are not within the list of selected 140 query-genes. This query-list consists of the overall top 140 mutated genes from all the selected pedcbioportal data.

We attempted to compare various subgroups of glioma diseases, however, the lack of meta-information regarding cancer type specificity of samples did not allow for sub-classification of LGG. Thus, future studies and additional data resources are necessary for a more detailed analysis. Still, the comparison of the distinct subgroups of HGG and LGG highlights the differences within the heterogeneous disease group of gliomas. Likewise, classification by grades I-IV would require more detailed meta-information or sample designation.

For future studies, it can be useful taking other variables into account in combination with age, such as analyzing fusion genes, gene expression, post-translational modifications depending on data availability or additional clinical data including therapy details.

## Conclusions

The idea of questioning known age groups in glioma classification offers new perspectives. Certain biomarkers are already associated with certain age groups. Changing age margins results in the movement of features to other age groups. These age-associated features resemble possible targets and biomarkers, that may lead to different diagnosis and treatment strategies. Nonetheless, it would be interesting to see better classifier difference when dealing with specific glioma subclasses. Therefore, future work based on the extension of this research requires additional glioma-grade-specific data to better compare specific glioma subtypes.

## Supplementary Information

**Additional file 1.** List of top mutated 140 genes used for query.

**Additional file 2.** Raw Mutation data downloaded from pedcbioportal and cbioportal, filtered, processed, merged and again filtered, provided as semicolon separated file as used in the python scripts.

## Data Availability

The data used in the present study are publicly available and have been downloaded from the public data repositories pedcbioportal and cbioportal. Sourcecode and processed data are available onhttps://github.com/radiance/glioma-mutations-xai.

## Abbreviations

ATRX: Alpha thalassemia/mental retardation syndrome X-linked chromatin remodeler
BCORL1: BCL6 corepressor like 1
BRAF: B-Raf proto-oncogene, serine/threonine kinase
CDKN2A/B: Cyclin dependent kinase inhibitor 2A or 2A
CTNNB1: Catenin beta 1
GBM: Glioblastoma multiform
H3F3A: H3 histone family member 3A
HIST1H3B/C: Histone cluster 1 H3 family member B or C
HGG: Higher grade glioma
IDH1: Isocitrate dehydrogenase 1
LGG: Lower grade glioma
RELA: RELA proto-oncogene, NF-KB subunit
SD: Standard deviation
SHAP: Shapley additive explanations
SHH: Sonic hedgehog signaling molecule
TCGA: The Cancer Genome Archive
TERT: Telomerase reverse transcriptase
TP53: Tumor protein P53
UI: User interface
WNT: Wingless-type MMTV integration site family
XAI: Explainable artificial intelligence
